# Lessons from Multicenter Studies on CSF Biomarkers for Alzheimer's Disease

**DOI:** 10.4061/2010/610613

**Published:** 2010-07-08

**Authors:** Niklas Mattsson, Henrik Zetterberg, kaj Blennow

**Affiliations:** The Clinical Neurochemistry Laboratory, Department of Psychiatry and Neurochemistry, Institute of Neuroscience and Physiology, Sahlgrenska University Hospital, The Sahlgrenska Academy at University of Gothenburg, 43180 Mölndal, Sweden

## Abstract

Several single-center studies have confirmed the usability of cerebrospinal fluid (CSF) biomarkers for the diagnosis of Alzheimer's disease (AD), even in early disease stages. Large scale multicenter studies have principally confirmed this, although such studies have also indicated the presence of significant intercenter and interlaboratory variations in biomarker measurements. Such variations may hamper the development of biomarkers and their introduction into clinical routine practice. Recently a quality control program run by the Alzheimer's Association was started in order to harmonize procedures of laboratories world-wide. This program provides both standardized guide lines and external control CSF samples, and will allow longitudinal evaluation of laboratory performance.

## 1. Introduction

The ongoing development of disease modifying treatment for Alzheimer's disease (AD) puts pressure on researchers to develop reliable biomarkers for diagnosis, disease progression and monitoring of treatment effect. For these purposes, the most promising biomarkers are imaging and cerebrospinal fluid (CSF) markers [1–3]. The core CSF biomarkers are *β*-amyoid42 (A*β*42), total-tau (T-tau), and phosphorylated tau (P-tau), where the most frequently used P-tau isoforms are tau phosphorylated at the threonine amino acid residues 181 or 231 [1]. CSF A*β*42, T-tau and P-tau correspond to the principal neuropathological elements of AD: extracellular amyloid plaques, axonal degeneration, and intraneuronal tangles. Indeed, autopsy studies and imaging studies have revealed that CSF biomarker concentrations correspond well to brain alterations [4–7]. The accessibility of CSF for analysis and the low cost of CSF biomarker measurements favor their usage for detection, and monitoring of pathological processes in the brain. Several studies have shown that AD patients have decreased CSF A*β*42 and increased T-tau and P-tau compared with healthy controls [8]. Noteworthy, T-tau and P-tau correlate in AD patients and controls but not in several other neurological diseases. T-tau is increased in several neurodegenerative conditions and is a sensitive but unspecific AD biomarker [9]. P-tau, on the other hand, may add specificity for AD in dementia investigations [10].

## 2. CSF Biomarkers in Early Diagnosis

Brain alterations are likely to start long before onset of clinical dementia. Thus, biomarkers that detect changes in the brain are likely altered at a clinically early stage. It has been proposed that biomarker alterations occur dynamically during the disease process, with A*β* and Tau markers changed first, followed by distortion of brain structure, decline of memory and ultimately clinical dysfunction [11]. Supporting the use of CSF biomarkers for early diagnosis, decreased A*β*42 and increased T-tau and P-tau are seen in patients with mild cognitive impairment (MCI) later diagnosed with AD. In a well-controlled monocenter study, Hansson and coworkers followed 137 MCI patients for 4–6 years, during which 57 patients were diagnosed with AD. The CSF biomarkers sampled at baseline had a sensitivity of 95% and a specificity 83% for incipient AD [12]. Since clinical AD diagnosis lacks some accuracy versus autopsy confirmation, it is probably not possible to achieve a significantly higher diagnostic accuracy for biomarkers towards clinical diagnosis. In line with this, consensus reports recommend that AD biomarkers should have at least 85% sensitivity and 75%–85% specificity [13]. As demonstrated by the study by Hansson and coworkers as well as other studies [14–20], this goal is achievable with the CSF biomarkers A*β*42, T-tau, and P-tau even in early stages.

## 3. Multicenter Studies of Diagnostic Accuracy

The results from the above mentioned studies are encouraging, but it should be noted that diagnostic biomarkers generally perform better in homogenous monocenter studies than in more challenging heterogeneous multicenter studies. So far, only a few large scale multicenter studies of CSF AD biomarkers have been published. In the DESCRIPA study, by Visser and coworkers, CSF samples were taken from 193 MCI and (subjective cognitive impairment) SCI patients and 89 controls at 9 centers in Europe [21]. All samples were analyzed at one laboratory. The results confirmed that a CSF AD profile, as defined by Hulstaert et al. [22], predicted AD in MCI with an odds ratio of 27 (95% CI 1.6–460) in amnestic MCI patients. However, the study was complicated by the fact that all controls were enrolled at one center, and CSF sampling procedures differed between centers. Also, 31% of healthy controls presented a CSF AD pattern, indicating a lack of specificity of the CSF biomarkers. This finding may seem controversial but is consistent with a by now large body of literature, showing that around one third of cognitively normal elderly display AD-like changes in their brains at autopsy [23], in their CSF [24, 25] or on PET scans imaging amyloid [26–28]. Longitudinal studies will tell us whether these individuals do have preclinical AD, or whether silent AD pathology is part of the normal aging process. Recent studies using repeated cognitive assessments [29] or functional magnetic resonance imaging measures of cortical network integrity [30] favor the first of the two scenarios.

Shaw and coworkers have published CSF biomarker results from the Alzheimer's Disease Neuroimaging Initiative (ADNI) [31]. The ADNI is a multicenter, longitudinal study, launched in 2004. In this study, optimal biomarker cut-offs where established in autopsy confirmed ADNI-independent AD patients and normal controls, with diagnostic accuracies ranging from 70% to 87%. The ADNI cohort was recruited at 56 clinical centers, which potentially could introduce large center-dependent variations. To minimize such variations, all participating centers followed the ADNI procedure manual. All samples were analyzed at the ADNI Biomarker Core laboratory at the University of Pennsylvania. The study included 196 MCI patients and 37 of these had been diagnosed with AD at the 12-month follow-up. A majority (87–89%) of these 37 presented a CSF AD profile at baseline. This similar to what was seen in patients with mild AD at baseline (N = 100, 88%–91%). However, as in the DESCRIPA study, a large proportion (34%–38%) of cognitively healthy controls (N = 114) had the same biomarker pattern, again indicating a lack of specificity in relation to clinical diagnosis.

In a third multicenter investigation, published in JAMA in 2009, we enrolled 750 MCI patients, 529 AD patients, and 304 healthy controls from 12 centers in Europe and the United States [32]. Four laboratories were involved, enabling evaluation not only of Intercenter, but also of interlaboratory differences, as discussed below. Cut-offs for the combination of A*β*42, T-tau, and P-tau were constructed in AD patients and controls, with sensitivity 85% in accordance with the above mentioned consensus document. This yielded 88% specificity towards healthy controls. In MCI patients followed for at least 2 years, the sensitivity of the biomarkers was 83% and the specificity 72%. The lower specificity compared to the monocenter study by Hansson et al. could partly be explained by the shorter followup, which makes it hard to verify the benign nature of all stable MCI cases. However, Intercenter variations may also have influenced the results and blurred some effects of the biomarkers. For the remaining part of this review, we will focus on such variations, and possible ways of eliminating them.

## 4. Intercenter Biomarker Variations

CSF studies on AD patients and controls report different biomarker concentrations, reference ranges, and diagnostic cut-offs [8]. CSF A*β*42 levels in AD patients in some studies even exceed the levels in controls in other studies. Such fluctuations make it hard or impossible to compare CSF biomarkers between centers and studies. These Intercenter variations come as no surprise. Rather, it is more rule than exception that a novel clinical chemical measurement present variations between centers, due to preanalytical or analytical confounding factors. Such variations are traditionally tackled by quality control programs, which until recently have been lacking for CSF dementia markers. For the CSF biomarkers A*β*42, T-tau, and P-tau, possible confounding factors include preanalytical, analytical, and assay factors [33–35]. Preanalytical factors include, for example, usage of different test tubes and differences in sample handling and storage. Analytical factors include differences in pipetting technique and other laboratory procedures. Finally, important assay factors are batch-to-batch variations and differences in standards and coating of antibodies. A growing number of laboratories are performing CSF analyses for dementia diagnostics. To facilitate the use of biomarkers in research settings and to enable their implementation in clinical routine, the Intercenter variations must be dealt with. Basic CSF parameters, including albumin and immunoglobulin levels, typically have interlaboratory coefficients of variation (CV) below 10%. This is a reasonable ultimate aim also for dementia markers. A handful of multicenter studies have investigated interlaboratory variations in CSF dementia markers, and we will now summarize the results of these studies.

## 5. The First International Quality Control Surveys

In 2006, Lewczuk and coworkers published the first international quality control survey of neurochemical dementia diagnostics [36]. A ventricular CSF sample was collected from a neurosurgical patient. Fourteen laboratories in Germany, Austria, and Switzerland participated and performed CSF biomarker measurements according to local routines. Different commercially available ELISAs were used for A*β* measurement. Three laboratories used a method from The Genetics Company (Zürich, Switzerland) measuring A*β*x-40 and A*β*x-42, with no specific N-terminal amino acid capture. Thirteen laboratories used the Innotest (Innogenetics, Ghent, Belgium) ELISA for A*β*, which specifically measures A*β*1-42. Fourteen laboratories used Innotest for T-tau, and 11 laboratories used Innotest for P-tau. Interlaboratory coefficients of variation ranged from 21% to 38%, with the highest numbers reported for A*β*x-42 (38%) and A*β*1-42 (29%). Most laboratories analyzed samples in duplicates and the intra-assay imprecision was mostly low, exceeding 5% only for T-tau (5.3%) and A*β*1-42 (7.5%).

Verwey and coworkers published another quality survey in 2009 involving 20 laboratories with measurements performed in 2004 and 2008, allowing evaluation of longitudinal stability [37]. For this study, large CSF pools with different biomarker patterns were constructed and samples were distributed to 13 laboratories in 2004 and to 18 laboratories in 2008. A majority of laboratories used the Innotest ELISA for T-tau and P-tau, but in the 2008 run some laboratories had introduced the Luminex method AlzBio3 (Innogenetics, Ghent, Belgium). ELISA methods generally give higher values for CSF biomarkers than Luminex methods, but the methods have equivalent diagnostic accuracy for AD [38, 39]. A*β*42 was measured with the Innotest ELISA, or ELISAs from The Genetics Company, Biosource (Invitrogen, Camarillo, USA) or an in-house assay. Three laboratories used the AlzBio3 method for A*β*42. Interlaboratory CVs were high for A*β*42 ELISAs in both 2004 (31%) and 2008 (37%), and somewhat lower when only including laboratories using the Innotest (30% and 22%, resp.). CVs were smaller for T-tau (2004: 21%, 2008: 16%) and P-tau (2004: 13%, 2008: 15%). The AlzBio3 method had CVs ranging from 14%–22%, but the low number of laboratories performing this assay makes interpretation difficult. Nine laboratories participated in both rounds of the survey and intra-laboratory CVs for these were 25% for A*β*42, 18% for T-tau and 7% for P-tau.

These two studies show large interlaboratory variations for A*β*42, and smaller but significant variations for T-tau and P-tau. Since these studies used centrally distributed control CSF, several preanalytical confounding factors were eliminated. Remaining possible causes of the variations include local differences in analytical routines, machinery differences and batch-to-batch variations in analytical kits. The latter provides a major challenge for kit vendors, and emphasizes the need to have a robust production of antibodies, standard solutions, and analytical plates.

## 6. The Swedish Brain Power Survey

In the multicenter study mentioned above, published in JAMA in 2009, most centers sent their samples to the Clinical Neurochemistry Laboratory in Mölndal for analysis. However, samples from Amsterdam, Munich, and Kuopio, Finland were analyzed at local laboratories. The study therefore provides information about interlaboratory differences. Subset of samples from Amsterdam, Munich and Kuopio were rerun at the laboratory in Mölndal, and values for A*β*42 and T-tau from all three local laboratories differed more than 2CVs from values measured in Mölndal, using Mölndal CVs for the assays (around 10%). However, even for centers where samples were run at the laboratory in Mölndal, considerable Intercenter variations were seen. Possible sources of these variations include the preanalytical procedures of subject selection, lumbar puncture, sample handling, and storage. Such variations were seen in particular for A*β*42, but to a less extent also for T-tau and P-tau.

## 7. The Alzheimer's Association Quality Control Program

The studies outlined above present clear evidence for Intercenter and interlaboratory variations in CSF biomarker measurements. This makes it difficult to compare studies, which may hamper development of CSF biomarkers. With the rapid development of disease modifying treatment the AD scientific community must not lose momentum in biomarker development. Standardization of collection and handling of samples is vital for this, such as suggested by, for example, the German Competence Net Dementias [40]. Accordingly, it was decided at the International Conference on Alzheimer's Disease (ICAD) in Vienna in 2009 to start an international quality control (QC) program for CSF AD biomarkers. This program is run by the Alzheimer's Association and administrated from the Clinical Neurochemistry Laboratory in Mölndal. The program is open for public, private, research, clinical, and pharmaceutical laboratories. Participating laboratories receive a chart with recommended guidelines for lumbar puncture and sample handling and storage. In line with the ADNI procedures manual, the intention of this chart is to harmonize local routines to eliminate confounding factors responsible for Intercenter variations. In a second part of the program, participating laboratories receive QC CSF samples, constructed in Mölndal. These are analyzed and results are reported to the QC program coordinator. The participating laboratories then receive feed-back on their analysis compared to the other laboratories. The first round of the program has just been completed and data are being analyzed. The QC program will continue with multiple rounds each year, enabling the tracking of longitudinal changes in performance.

## 8. Conclusions

Multicenter studies have confirmed the high diagnostic accuracy of CSF biomarkers for AD, even at early stage, before onset of dementia. In particular, the high diagnostic sensitivity of CSF biomarkers achieved in the ADNI trial shows that harmonization of sample collection and handling allows the usage of the biomarkers even in a wide-spread multicenter setting. This advocates the use of CSF biomarkers in clinical studies, where they may be used to enrich trials with MCI patients with incipient AD. However, to facilitate the development of biomarkers and to enable their introduction in clinical routine, interlaboratory and Intercenter differences should be systematically analyzed. This is achievable within the Alzheimer's Association QC program.
